# SNAP-25 is a promising novel cerebrospinal fluid biomarker for synapse degeneration in Alzheimer’s disease

**DOI:** 10.1186/1750-1326-9-53

**Published:** 2014-11-23

**Authors:** Ann Brinkmalm, Gunnar Brinkmalm, William G Honer, Lutz Frölich, Lucrezia Hausner, Lennart Minthon, Oskar Hansson, Anders Wallin, Henrik Zetterberg, Kaj Blennow, Annika Öhrfelt

**Affiliations:** Institute of Neuroscience and Physiology, Department of Psychiatry and Neurochemistry, Sahlgrenska Academy at the University of Gothenburg, S-431 80 Mölndal, Sweden; Department of Psychiatry, University of British Columbia, Vancouver, Canada; Department of Geriatric Psychiatry, Central Institute for Mental Health Mannheim, University of Heidelberg, Mannheim, Germany; Clinical Memory Research Unit, Department of Clinical Sciences, Lund University, Lund, Sweden; Memory Clinic, Skåne University Hospital, Skåne, Sweden; UCL Institute of Neurology, Queen Square, London, WC1N 3BG London, UK

**Keywords:** Alzheimer’s disease, Biomarker, Cerebrospinal fluid, SNAP-25, SNARE proteins, Mass spectrometry, Immunopurification, Selected reaction monitoring

## Abstract

**Background:**

Synaptic degeneration is an early pathogenic event in Alzheimer’s disease, associated with cognitive impairment and disease progression. Cerebrospinal fluid biomarkers reflecting synaptic integrity would be highly valuable tools to monitor synaptic degeneration directly in patients. We previously showed that synaptic proteins such as synaptotagmin and synaptosomal-associated protein 25 (SNAP-25) could be detected in pooled samples of cerebrospinal fluid, however these assays were not sensitive enough for individual samples.

**Results:**

We report a new strategy to study synaptic pathology by using affinity purification and mass spectrometry to measure the levels of the presynaptic protein SNAP-25 in cerebrospinal fluid. By applying this novel affinity mass spectrometry strategy on three separate cohorts of patients, the value of SNAP-25 as a cerebrospinal fluid biomarker for synaptic integrity in Alzheimer’s disease was assessed for the first time. We found significantly higher levels of cerebrospinal fluid SNAP-25 fragments in Alzheimer’s disease, even in the very early stages, in three separate cohorts. Cerebrospinal fluid SNAP-25 differentiated Alzheimer’s disease from controls with area under the curve of 0.901 (*P* < 0.0001).

**Conclusions:**

We developed a sensitive method to analyze SNAP-25 levels in individual CSF samples that to our knowledge was not possible previously. Our results support the notion that synaptic biomarkers may be important tools for early diagnosis, assessment of disease progression, and to monitor drug effects in treatment trials.

**Electronic supplementary material:**

The online version of this article (doi:10.1186/1750-1326-9-53) contains supplementary material, which is available to authorized users.

## Background

Animal models of the early phases of Alzheimer’s disease have directly demonstrated loss of presynaptic proteins and synaptic dysfunction [1, 2]. In patients, however, assays for presynaptic proteins are indirect or rely on post-mortem findings. In the early stages of disease they have provided inconsistent results reporting elevated, unchanged, and lower protein amounts [3–12]. Biomarker studies of amyloidβ1-42 (Aβ1-42), total tau (T-tau) and tau phosphorylated at threonine 181 (P-tau_181_) in cerebrospinal fluid (CSF) have contributed to understanding the sequence of clinically relevant molecular events contributing to cognitive impairment [13]. In the central nervous system synaptosomal-associated protein 25 (SNAP-25) is an important marker of functional synapses, being one essential component of the soluble N-ethylmaleimide-sensitive factor attachment protein receptors (SNARE) complex. These proteins mediate synaptic communication by initiating fusion of synaptic vesicles [14].

The notions that synaptic loss occurs early in Alzheimer’s disease, and that synaptic proteins at active synapses could be biomarkers indicating the degree of synaptic degeneration have prompted interest in detecting relevant synaptic proteins in human biological fluid samples. Analysis of synaptic proteins in CSF is complicated by the presence of only trace amounts, and the membrane-bound nature of many of these proteins [15]. Several research groups, including our own, have detected synaptic proteins in CSF [16–21]. However, these studies were performed on relatively large quantities of pooled CSF from multiple patients [16, 17, 21]. Moreover, the target proteins had to be selectively purified and concentrated in several steps and the quantitative aspects of the techniques may have been sub-optimal [20].

Here, we have developed an assay where the concentration of the presynaptic protein SNAP-25 could be reproducibly measured in CSF samples from individual patients. We hypothesized that soluble forms of brain SNAP-25 were the most likely to resemble SNAP-25 in CSF. Since SNAP-25 is abundant in brain tissue we used biochemically fractionated human brain homogenate (soluble, membrane-bound, and membrane-raft associated protein fractions) to design a strategy for quantification of SNAP-25 in CSF by combining selective purification with immunoprecipitation, digestion with trypsin, and mass spectrometry analysis. We found significantly higher levels of SNAP-25 in CSF in Alzheimer’s disease in three separate cohorts, including in the very early stage of the disease.

## Results

### Characterization and quantification of SNAP-25 in human brain tissue

**Figure 1:**
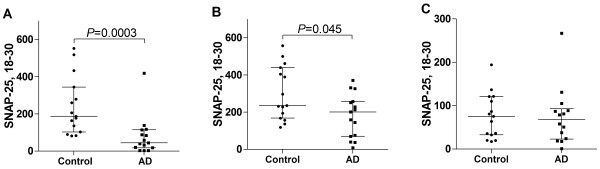
**Targeted SRM-MS analyses of SNAP-25 in human brain.** Individual values for the SRM-MS measured ratios (endogenous peptide/labeled peptide standard) of immunoprecipitated [antibodies SP12 **(A-B)** and SMI81 **(C)** SNAP-25, in biochemically fractionated membrane-bound **(A)** and membrane-raft associated **(B)** and soluble **(C)**, extract of superior parietal gyrus from controls (N = 15) and patients with Alzheimer’s disease (N = 15). The lower, upper and middle lines of the error bars correspond to the 25th and 75th percentiles and medians, respectively. SMI81 recognizes the extreme N-terminus of SNAP-25, especially when it’s N-terminal acetylated. The exact epitope of SP12 is unknown.

We used the monoclonal antibodies to affinity purify SNAP-25 from biochemically fractionated human brain homogenate. Using the SP12 antibody and quantification with selected reaction monitoring mass spectrometry (SRM-MS) we compared the SNAP-25 levels in brain homogenate fractions from Alzheimer’s disease patients (N = 15) and age-matched controls (N = 15) (Additional file 1: Table S1). We found that the levels of SNAP-25 were significantly lower in the Alzheimer’s disease group for the membrane-bound and the membrane-raft associated fractions (Figure 1A-B). In contrast, the levels of SNAP-25 in the soluble protein fractions were very low or undetectable (data not shown). However, when affinity purifying with SMI81 instead of SP12, SNAP-25 could be quantified in all soluble fractions (Figure 1C). Nevertheless, no significant differences between the levels of soluble SNAP-25 in Alzheimer’s disease and control brain homogenate samples were observed.

**Figure 2:**
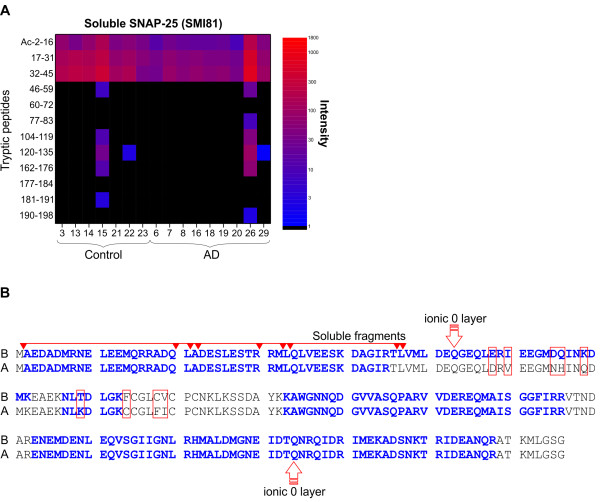
**Sequence coverage of SNAP-25. (A)** Heat map for the relative LC-MS signal intensities of individual tryptic peptides from SNAP-25B. Biochemically fractionated soluble proteins of superior parietal gyrus from controls (N = 7) and patients with Alzheimer’s disease (N = 9) were immunoprecipitated with SMI81. High resolution LC-MS ion chromatograms of all peptides identified as belonging to SNAP-25B were extracted. An increase of signal intensity is seen as a blue to red shift. **(B)** Amino acid sequences for SNAP-25B and SNAP-25A. Soluble endogenous forms identified by top-down LC-MS/MS analysis are indicated by arrows above the sequences. Differences between SNAP-25B and SNAP-25A are indicated by boxes. Amino acids belonging to tryptic peptides identified by LC-MS/MS are labeled bold. The two glutamines (Q) indicated “ionic 0 layer” contribute together with a syntaxin glutamine (Q), and a VAMP arginine (R) to the highly conserved 3Q:1R motif at the core of the SNARE complex.

To investigate if the dissimilar levels of SP12- and SMI81-immunoreactive SNAP-25 in the soluble fractions could be due to a truncation or other post-translational modifications we analyzed a subset of the samples with LC-MS/MS. In the majority of the soluble fractions, only tryptic peptides originating from the N-terminal part of the SNAP-25 protein were detected (Figure 2), indicating that the soluble SNAP-25 were C-terminally truncated. However, in all the membrane-bound and membrane-raft associated fractions the entire SNAP-25 protein was detected (regardless of antibody) (Additional file 1: Figure S1). Moreover, in all fractions, including the soluble, SNAP-25 was N-terminally modified by methionine excision and acetylation.

To characterize the soluble SNAP-25 forms we used a top-down LC-MS/MS approach. Using undigested proteins from the SMI81 affinity purified soluble fraction, we successfully identified eight truncated forms of soluble SNAP-25, all N-terminally modified by methionine excision and acetylation (Additional file 1: Figure S2, Table S2). Building on the findings of the soluble truncated SNAP-25 peptides we changed our approach to target the furthermost N-terminal tryptic peptides of SNAP-25 (Figure 2B) and perform the MS-based quantification with high resolution selected ion monitoring (HR-SIM-MS) on a Quadrupole-Orbitrap Mass Spectrometer (Q Exactive) [22]. The reproducibility of the novel method was measured and the CV of the SNAP-25 levels was found to be less than 10% (Additional file 1: Table S3).

### Evaluation of SNAP-25 as a synaptic marker in CSF samples

#### Demographic results

Table 1 shows the demographic characteristics of the groups. The German cohort was composed of nine patients with Alzheimer’s disease (three men and six women, 62-83 years), seven subjects with prodromal Alzheimer’s disease (four men and three women, 57-77 years), and nine non-demented controls (two men and seven women, 60-83 years). The first replication set (Swedish cohort I) was composed of 10 patients with Alzheimer’s disease (three men and seven women, 59-84 years), and six non-demented controls (one man and five women, 47-64 years). The second replication set (Swedish cohort II) was composed of 17 patients with Alzheimer’s disease (five men and 12 women, 61-76 years), and 17 healthy controls (nine men and eight women, 63-70 years). The patients and controls in the German and Swedish cohort II were age-matched, while the patients with Alzheimer’s disease were significantly older than the controls in the Swedish cohort I. In all three cohorts, patients with Alzheimer’s disease had a significantly lower MMSE score compared with the controls.

**Table 1:** **Demographic data and biomarker CSF levels for the diagnostic groups** ^**a**^

German cohort	Control	Prodromal Alzheimer’s disesase	Alzheimer’s disease
Number (Men/Women)	9 (2/7)	7 (4/3)	9 (3/6)
Age (years)	70 (68-74)	72 (69-73)	68 (68-79)
MMSE	27 (25-29)	28 (27-28)	22 (21-23) *P* = 0.02^b^, *P* = 0.001^c^
Aβ1-42 (ng/L)	1065 (797-1201)	541 (521-753) *P* = 0.005^b^	524 (424-695) *P* = 0.0005^b^
T-tau (ng/L)	165 (135-208)	403 (353-513) *P* = 0.0002^b^	779 (683-864) *P* = 0.00004^b^, *P* = 0.002^c^
P-tau_181_ (ng/L)	45 (38-50)	81 (76-95) *P* = 0.0002^b^	130 (108-161) *P* = 0.00004^b^, P = 0.005^c^
**Swedish cohort I**	**Control**	**Prodromal Alzheimer’s disesase**	**Alzheimer’s disease**
Number (Men/Women)	6 (1/5)		10 (3/7)
Age (years)	54 (48-63)		77 (73-82) *P* = 0.001^b^
MMSE	27 (27-28)		24 (22-25) *P* = 0.0003^b^
Aβ1-42 (ng/L)	915 (860-1040)		470 (355-560) *P* = 0.0003^b^
T-tau (ng/L)	290 (230-300)		690 (590-1100) *P* = 0.0002^b^
P-tau_181_ (ng/L)	56 (40-60)		92 (84-132) *P* = 0.0003^b^
**Swedish cohort II**	**Control**	**Prodromal Alzheimer’s disesase**	**Alzheimer’s disease**
Number (Men/Women)	17 (9/8)		17 (5/12)
Age (years)	66 (64-68)		68 (66-70)
MMSE	30 (29-30)		25 (24-27) *P* < 0.0001^b^
Aβ1-42 (ng/L)	640 (530-870)		320 (210-440) *P* = 0.0001^b^
T-tau (ng/L)	250 (180-320)		560 (360-1020) *P* = 0.0006^b^
P-tau_181_ (ng/L)	46 (34-58)		93 (54-127) *P* = 0.001^b^

*Abbreviations*: Aβ1-42 (amyloidβ 1-42), CSF (cerebrospinal fluid), MMSE (mini-mental state examination), T-tau (total tau), P-tau_181_ (tau phosphorylated at threonine 181).

^a^Data are given as median (interquartile range) unless otherwise indicated. Statistical differences were determined using nonparametric tests.

^b^Compared with controls.

^c^Compared with prodromal Alzheimer’s disease.

### Levels of SNAP-25 in CSF

**Figure 3:**
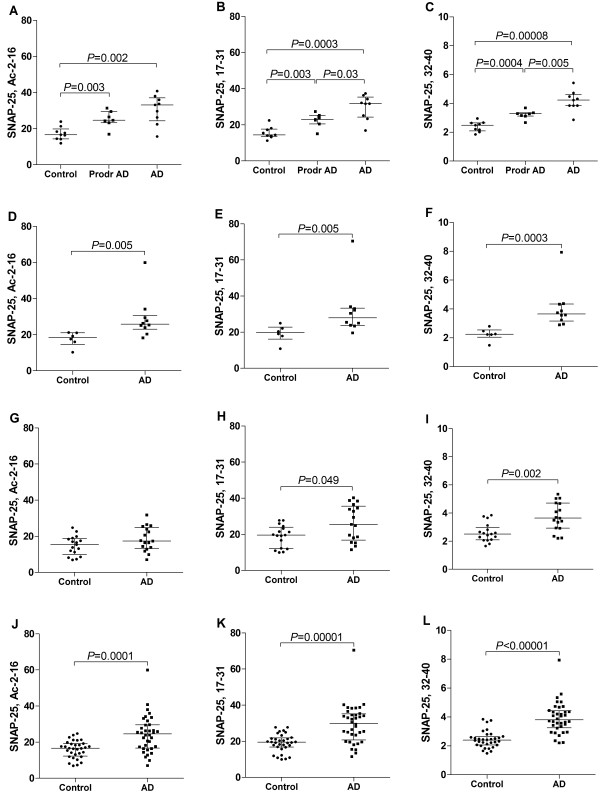
**Targeted HR-SIM-MS analyses of SNAP-25 in human CSF.** Individual values for the HR-SIM-MS measured peak area ratios ([endogenous peptide/labeled peptide standard] multiplied by 10,000) of immunoprecipitated (SMI81 antibody) SNAP-25 in CSF samples within German cohort **(A-C)**, Swedish cohort I **(D-F)**, Swedish cohort II **(G-I)**, and the entire group of Alzheimer’s disease (N = 36) and controls (N = 32) **(J-L)**. The nine panels depict the measured levels of three N-terminal tryptic peptides of SNAP-25, Ac-2-16 **(A, D, G, J)**, 17-31 **(B, E, H, K)**, and 32-40 **(C, F, I, L)**.

The CSF levels of all three investigated tryptic peptides of SNAP-25 were significantly higher in patients with prodromal Alzheimer’s disease and overt Alzheimer’s disease compared with non-demented controls (Figure 3A-C). Moreover, two of the SNAP-25 peptides (amino acids 17-31 and 32-40) were significantly higher in Alzheimer’s disease compared with prodromal Alzheimer’s disease (Figure 3B-C). Consistently, the CSF levels of all tryptic peptides of SNAP-25 were significantly higher in patients with Alzheimer’s disease compared with non-demented controls in the replication set (Figure 3D-F). In the second replication set, the CSF levels of the tryptic peptide of SNAP-25 (32-40) were significantly higher in patients with Alzheimer’s disease compared with healthy controls (Figure 3I).

**Figure 4:**
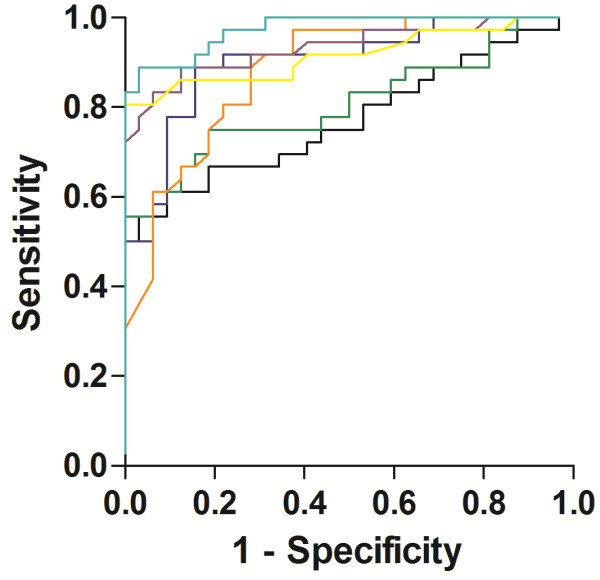
**ROC curve analysis of SNAP-25 in human CSF.** ROC curve analysis for SNAP-25 32-40 (turquoise), SNAP-25 17-31 (green), SNAP-25 Ac-2-16 (black) Aβ1-42 (orange), T-tau (purple) and P-tau_181_ (yellow) in CSF for differentiation of Alzheimer’s disease patients from controls in the entire subject material. The area under the curve (95% confidence interval) was 0.901 (0.828-0.974) (*P* < 0.0001), 0.808 (0.703-0.913) (*P* < 0.0001), 0.772 (0.659-0.885) (*P* < 0.001), 0.881 (0.802-0.960) (*P* < 0.0001), 0.933 (0.873-0.994) (*P* < 0.0001), and 0.916 (0.844-0.987) (*P* < 0.0001), respectively.

There were no cohort effects on the CSF levels of novel SNAP-25 biomarkers, P-tau_181_ or Aβ1-42 (data not shown), allowing statistical analyses of the entire group of participant samples from Alzheimer’s disease (N = 36) and controls (N = 32) (Figure 3J-L). The tryptic peptide assays of SNAP-25 (32-40, 17-31, and Ac-2-16) and CSF biomarkers (Aβ1-42, T-tau and P-tau_181_) could each differentiate Alzheimer’s disease (N = 36) from controls (N = 32), with area under the curve of 0.901 (0.828-0.974) (*P* < 0.0001), 0.808 (0.703-0.913) (*P* < 0.0001), 0.772 (0.659-0.885) (*P* < 0.001), 0.881 (0.802-0.960) (*P* < 0.0001), 0.933 (0.873-0.994) (*P* < 0.0001), and 0.916 (0.844-0.987) (*P* < 0.0001), respectively (Figure 4).

No statistically significant correlations between age and levels of the tryptic peptides of SNAP-25 (Ac-2-16, 17-31, and 32-40) were observed in either the control group (N = 33) or the Alzheimer’s disease group (N = 36) (Table 2). The MMSE score, indicating the severity of cognitive impairment, correlated significantly with SNAP-25 (Ac-2-16) in the Alzheimer’s disease (N = 36) group (Table 2). All tryptic peptides of SNAP-25 (Ac-2-16, 17-31, and 32-40) correlated with the levels of T-tau and P-tau_181_ in both the control group (N = 33) and in patients with Alzheimer’s disease (N = 36) (Table 2). The tryptic peptides of SNAP-25 (Ac-2-16 and 17-31) correlated with Aβ1-42 in the control group, but not in patients with Alzheimer’s disease (Table 2).

**Table 2:** **Correlation between age, MMSE and biomarker levels in the entire CSF material** ^**a**^

	SNAP-25, Ac-2-16	SNAP-25, 17-31	SNAP-25, 32-40
Cont (N = 33)			
Age	N.S.	N.S.	N.S.
MMSE	N.S.	N.S.	N.S.
Aβ1-42	rho = 0.560, *P* = 0.001	rho = 0.468, *P* = 0.007	N.S.
T-tau	rho = 0.564, *P* = 0.001	rho = 0.563, *P* = 0.001	rho = 0.656, *P* = 0.0001
P-tau_181_	rho = 0.735, *P* < 0.0001	rho = 0.715, *P* < 0.0001	rho = 0.659, *P* < 0.0001
SNAP-25, Ac-2-16	-	rho = 0.859, *P* < 0.0001	rho = 0.718, *P* < 0.0001
SNAP-25, 17-31	-	-	rho = 0.799, *P* < 0.0001
AD (N = 36)			
Age	N.S.	N.S.	N.S.
MMSE	rho = -0.509, *P* = 0.002	N.S.	N.S.
Aβ1-42	N.S.	N.S.	N.S.
T-tau	rho = 0.557, *P* = 0.0004	rho = 0.732, *P* < 0.0001	rho = 0.824, *P* < 0.0001
P-tau_181_	rho = 0.717, *P* < 0.0001	rho = 0.808, *P* < 0.0001	rho = 0.835, *P* < 0.0001
SNAP-25, Ac-2-16	-	rho = 0.836, *P* < 0.0001	rho = 0.720, *P* < 0.0001
SNAP-25, 17-31	-	-	rho = 0.900, *P* < 0.0001

*Abbreviations*: Aβ1-42 (amyloidβ 1-42), AD (Alzheimer’s disease), CSF (cerebrospinal fluid), MMSE (mini-mental state examination), T-tau (total tau), P-tau_181_ (tau phosphorylated at threonine 181).

^a^Correlations presented by the Spearman’s rank correlation coefficient (rho). Non significant (N.S.) (P-value > 0.05) correlations were not reported.

## Discussion

We report a new strategy to study synaptic pathology by using affinity purification and quantitative mass spectrometry to measure levels of the presynaptic SNAP-25 in CSF samples from individual patients. This is the first study demonstrating that SNAP-25 might be a useful CSF biomarker in differential diagnosis of patients with Alzheimer’s disease/prodromal Alzheimer’s disease from controls and also in discriminating Alzheimer’s disease from prodromal Alzheimer’s disease.

The novel CSF SNAP-25 assay was developed using biochemically fractionated human brain homogenate with high concentration of synaptic proteins. We found that the levels of SNAP-25 were significantly lower in the Alzheimer’s disease group for the membrane-bound and the membrane-raft associated fractions. These results are consistent with previous studies of SNAP-25 in Alzheimer’s disease using different immunoassays [5, 6, 8, 23, 24] and demonstrated that the method was sufficiently sensitive to detect pathological changes in brain tissue samples from individual patients.

The dissimilar levels of SP12- and SMI81-immunoreactive SNAP-25 in the soluble fractions made us hypothesize that soluble SNAP-25 may be truncated or modified compared to membrane associated SNAP-25. We found that only tryptic peptides originating from the N-terminal part of the soluble SNAP-25 protein were detected. Moreover, in all fractions, including the soluble, SNAP-25 was N-terminally modified by methionine excision and acetylation, consistent with a previous study [25]. The existence of a soluble N-terminal peptide fragment of SNAP-25 is consistent with the observations that immunoprecipitation with a monoclonal antibody directed towards the N-terminal of SNAP-25 increases the yield.

Truncated soluble forms of SNAP-25 had never been reported before and we characterized the various forms with a top-down tandem MS approach. Undigested proteins from the SMI81 affinity purified soluble fraction were analyzed directly on LC-MS/MS. We successfully identified eight truncated forms of soluble SNAP-25, all N-terminally modified by methionine excision and acetylation. An interesting finding was that the potential cleavage site for the creation of the longest truncated form of SNAP-25 (Ac-2-47) is located very close to the ionic zero layer at the center of the SNARE complex [26, 27] (Figure 2B). However, the longest soluble SNAP-25 contains the N-terminal amino acids 2-47 of the protein while the two isoforms SNAP-25A and SNAP-25B differs in amino acids 58, 60, 65, 69, 79, 84, and 88-89. Hence, soluble SNAP-25 no longer contains information regarding its original isoform (Figure 2B).

We found that the truncated forms of SNAP-25 were present in CSF, and the level of the tryptic peptide of SNAP-25 (32-40) was consistently and significantly higher in patients with Alzheimer’s disease compared with controls in the three independent cohorts. The tryptic peptide assays of SNAP-25 (32-40, 17-31, and Ac-2-16) could each differentiate Alzheimer’s disease from controls. However, the tryptic peptide assay of SNAP-25 (32-40) provided a slightly better differentiation of patients with Alzheimer’s disease from controls compared with the tryptic assays of SNAP-25 (Ac-2-16 and 17-31). The CSF levels of two of the tryptic peptides of SNAP-25 (Ac-2-16 and 17-31) were significantly higher in patients with Alzheimer’s disease compared with controls in two of the three examined clinical cohorts. Summarizing, these findings suggest SNAP-25 (32-40) to provide the best differential diagnostic biomarker of Alzheimer’s disease and showed differentiation of patients with Alzheimer’s disease from controls in a similar magnitude as the CSF biomarkers (Aβ1-42, T-tau and P-tau_181_).

In the early stages of disease, synaptic markers in previous studies provided inconsistent results reporting elevated, unchanged, and lower protein amounts [3–12]. In the present study, CSF SNAP-25 peptides were already increased in prodromal Alzheimer’s disease compared with controls, supporting the notion that this synaptic marker might provide an early marker for Alzheimer’s disease. Two of the SNAP-25 peptides (17-31 and 32-40) could also be used to differentiate prodromal Alzheimer’s disease and overt Alzheimer’s disease. A limitation of this study is the small sample size for patients with mild cognitive impairment, therefore the value of SNAP-25 as an early biomarker remains to be established.

The patients and controls in two of the cohorts were age-matched, while the patients with Alzheimer’s disease were significantly older than the controls in the Swedish cohort I. However, no statistically significant correlations between age and levels of the tryptic peptides of SNAP-25 (Ac-2-16, 17-31, and 32-40) in either the control group or the Alzheimer’s disease group were observed, suggesting that the detected SNAP-25 fragments not are influenced by age.

To date there is no CSF biomarker available that makes it possible to follow the progression of cognitive decline. Previous studies suggest that synaptic loss correlates with the clinical manifestations of Alzheimer’s disease, while there is no relation between the number of accumulated parenchymal amyloid plaques and synaptic pathology [3, 4]. In the present study, there was a negative correlation between SNAP-25 (Ac-2-16) and the MMSE, indicating patients suffering from more severe cognitive decline had higher levels of SNAP-25 (Ac-2-16), which implies that the novel biomarker might be useful to follow progression of cognitive decline. Interestingly, previous studies have shown evidence that *SNAP-25* single nucleotide polymorphisms are associated with cognitive decline [28, 29].

The CSF level of T-tau generally reflects the intensity of axonal and neuronal degeneration occurring in brain, while P-tau_181_ serves as a more specific marker for Alzheimer’s disease [30] CSF T-tau, P-tau_181_ and Aβ1-42 are stable over time making these Alzheimer’s biomarkers feasible for monitoring biochemical effects in clinical trials [31]. The finding that all investigated SNAP-25 peptides correlated well with T-tau and P-tau_181_, suggests that SNAP-25 might be a useful as a surrogate biomarker in future clinical treatment studies with tau modifying drugs [32].

## Conclusions

In summary, we have developed an assay allowing reproducible measurement of the level of the presynaptic protein SNAP-25 in CSF samples from individual patients. We demonstrate significantly higher levels of SNAP-25 in CSF samples from patients with prodromal Alzheimer’s disease and Alzheimer’s disease compared with controls. Our results show that SNAP-25 is a promising novel CSF biomarker for synapse degeneration in Alzheimer’s disease. This finding could be important for earlier diagnosis, assessment of progression of disease and to monitor drug effects in treatment trials in neurodegenerative diseases. We also report the identification of previously unknown, truncated soluble forms of SNAP-25 that could be employed to study the dynamics of SNARE protein processing and recycling.

## Methods

### Human brain tissue samples

The study included autopsy-confirmed patients with Alzheimer’s disease (N = 15) and age-matched controls (N = 15). Brain tissues from the region superior parietal gyrus were analyzed. All brain tissues were obtained from the Netherlands Brain Bank. Braak and Braak criteria, which are based on the distribution of neurofibrillary tangles, were used to categorize the stage of Alzheimer’s disease [33]. All Alzheimer’s disease patients fulfilled Braak stages 5 or 6, while the controls fulfilled Braak stages 0 or 1. Additional file 1: Table S1 shows the clinical and demographic characteristics of the groups.

### CSF samples

The exploratory phase of the investigation was performed on pooled decoded CSF samples supplied by the Clinical Neurochemistry Laboratory, Sahlgrenska University Hospital Sweden, from patients who underwent lumbar puncture to exclude infectious disorders of the central nervous system.

### The German cohort

CSF samples were obtained at the Interdisciplinary Memory Clinic of the Department of Geriatric Psychiatry of the Clinic of Psychiatry at the Central Institute of Mental Health, Mannheim from subjects with Alzheimer’s disease (N = 9), prodromal Alzheimer’s disease (N = 7) and non-demented controls (N = 9) (Table 1). Alzheimer’s disease was diagnosed according to the NINCDS-ADRDA criteria, with all Alzheimer’s disease patients fulfilling the criteria for probable Alzheimer’s disease [34]. Mild cognitive impairment due to Alzheimer’s disease was diagnosed according the new research criteria of Albert *et al* in 2011 [35]. Mild cognitive impairment was considered due to prodromal Alzheimer’s disease if additionally, biomarkers of molecular neuropathology of Alzheimer’s disease in CSF were measured positively for Alzheimer’s disease (CSF biomarkers Aβ1-42 ≤450 ng/L; T-tau ≥450 ng/L; P-tau_181_ ≥61 ng/L) or if there was hippocampal volume reduction or medial temporal atrophy assessed by visual rating (Schelten’s scale >2) measured by an experienced neuroradiologist. Non-demented controls had various psychiatric diagnoses, (including geriatric depression, and schizophrenia), Lumbar puncture in these patients was carried out for clinical indications, such as excluding organic brain disorder. All were found normal on cognitive screening tests, all routine CSF analyses were within normal limits and none of the CSF biomarkers were positive for Alzheimer’s disease.

### The Swedish cohort I

CSF samples were obtained at the Memory Clinic at Skåne University Hospital in Malmö from subjects with Alzheimer’s disease (N = 10) and non-demented controls (N = 6) (Table 1). Subjects diagnosed with Alzheimer’s disease met the DSM-III-R criteria for dementia [36] and the criteria for probable Alzheimer’s disease, as defined by NINCDS-ADRDA [34]. The non-demented cases exhibited cognitive complaints, but did not fulfil the criteria for dementia. To rule out preclinical Alzheimer’s disease in the latter group we only included cases with normal CSF Aβ1-42 > 550 ng/L and T-tau <400 ng/L levels.

### The Swedish cohort II

CSF samples from subjects with Alzheimer’s disease (N = 17) and healthy controls (N = 17) were obtained from the Gothenburg mild cognitive impairment study for which the diagnostic procedure was described in detail previously [37] (Table 1). The diagnosis of dementia was based on the DSM-III-R criteria [36] together with the criteria of NINCDS-ADRDA [34] and ICD-10 [38] with regard to Alzheimer’s disease. Controls were not included if they had subjective or objective signs of a cognitive disorder.

### CSF collection

All CSF samples were obtained by lumbar puncture though the L3/L4 interspace. The CSF samples were centrifuged at 2,000 g for 10 min at room temperature to remove cells and debris, and stored in aliquots at –80°C pending biochemical analysis.

### Homogenization of brain tissue

The brain extraction procedure was performed as described by Öhrfelt *et al*. with minor modifications [39]. Briefly, 100 ± 10 mg of brain tissue was homogenized on ice in 1 mL Tris- hydrochloride buffer (10 mM Tris-HCl, pH 6.8) containing complete protease inhibitor (Roche Diagnostics GmBH). Centrifugation of the homogenate was performed at 31,000 g for 1 h at +4°C and the supernatant was collected (Tris fraction, i.e., soluble fraction). One milliliter of Tris-buffer containing 0.5% Triton X-100 (Union Carbide Corporation) with complete protease inhibitor was added, and the pellet was homogenized on ice and sonicated using a micro-probe sonicator. The centrifugation step was repeated and the supernatant was collected (0.5% Triton fraction, i.e., membrane-bound fraction). The same procedure was repeated by addition of Tris-buffer containing 2% Triton (2% Triton fraction) and complete protease inhibitor, and again by addition of Tris-buffer containing 0.5% sodium dodecyl sulphate and complete protease inhibitor for a final centrifugation at +12°C (SDS fraction, i.e., membrane-raft associated fraction). All supernatants were aliquoted and stored at –80°C pending analysis. For protein quantitation, Protein DC assay (Bio-Rad Laboratories) reagent was used. This reagent contains a reducing agent and is detergent compatible.

### Analysis of CSF biomarkers

The CSF analyses on Aβ1-42, T-tau and P-tau_181_ levels were performed using commercially available assays from Fujirebio (INNOTEST® β-AMYLOID_(1-42)_, INNOTEST® hTAU Ag and INNOTEST® PHOSPHO-TAU(181P).

### Antibodies and recombinant protein of SNAP-25

The following antibodies were used: mouse monoclonal antibody SP12 recognizing SNAP-25 [40, 41] and mouse monoclonal antibody SMI81 (Covance) against SNAP-25 [25]. The SMI81 antibody recognizes an epitope containing the N-terminally acetylated first 11 amino acids of brain SNAP-25 [25]. Recombinant standard protein of SNAP-25 was purchased from Origene.

### Immunoprecipitation

The immunoprecipitation method for brain tissue extracts and CSF samples was performed according to Öhrfelt *et al*. with minor modifications [39]. Briefly, an aliquot (1 μg) of the mouse monoclonal antibody SP12 (1 g/L) or the mouse monoclonal antibody SMI81 (1 g/L) or IgG from murine serum (1 g/L, Sigma-Aldrich) (a negative control), was separately added to 100 μL magnetic Dynabeads M-280 Sheep anti-mouse IgG (Invitrogen Corporation) and incubated 1 h on a rocking platform at room temperature. The beads were washed three times with 1 mL of PBS (10 mM Na-phosphate, 0.15 M NaCl, pH 7.4). The antibodies were cross-linked using 20 mM dimethyl pimelimidate dihydrochloride (Sigma-Aldrich) and 0.2 M triethanolamine (pH 8.2 Sigma-Aldrich) according to the manufacturer’s product description. The cross-linked beads were washed two times in PBS and were blocked with Roti-Block (Carl Roth) for 1 h on a rocking platform at room temperature. Each brain tissue extract (soluble, membrane-bound, and membrane-raft associated proteins) (26 μg of total protein) and CSF samples (German cohort, 890 μL; Swedish cohort I, 700 μL; Swedish cohort II, 600 μL) were adjusted with 20% Triton and PBS to a final concentration of 0.2% Triton and a final volume of 1 mL). Samples and magnetic beads were incubated overnight on a rocking platform at +4°C. The magnetic beads/sample solution was transferred to the KingFisher magnetic particle processor (Thermo Fisher Scientific), tube 1. The following three wash steps (tubes 2-4) were conducted for 10 s in 1 mL of each washing buffer: (tube 2) 0.025% Tween 20 in PBS, (tube 3) PBS and (tube 4) 50 mM ammonium hydrogen carbonate (NH_4_HCO_3_, pH 8.0). SNAP-25 was then eluted from the beads by adding 100 μL 0.5% formic acid (FA) (tube 5) for 4 min. The eluted fractions were transferred to 0.5 mL Protein LoBind Tube (Eppendorf AG) and dried in a vacuum centrifuge.

### Protein digestion and addition of heavy-isotope labeled peptide standards

#### Brain tissue

The dried immunoprecipitated brain tissue samples were dissolved in 10 μL 0.1% RapiGest SF Surfactant (Waters) in 50 mM NH_4_HCO_3_ 1 h in room temperature. Disulfide bonds were reduced by addition of 10 μL 10 mM dithiothreitol (Sigma-Aldrich) in 50 mM NH_4_HCO_3_ and incubation for 3 min at +90°C. After cooling to room temperature, 5 μL 10 mM iodoacetamide (Sigma-Aldrich) in 50 mM NH_4_HCO_3_ was added, and the samples were incubated in the dark at room temperature for 30 min. Digestion was carried out by adding 5 μL trypsin solution (1 μg Sequencing Grade Modified Trypsin [Promega] dissolved in 0.01% aqueous HCl [0.1 g/L] and diluted to 5 mg/L in 50 mM NH_4_HCO_3_) and incubating overnight at +37°C. To reduce the amount of RapiGest SF Surfactant in the samples 2 μL 10% aqueous trifluoroacetic acid was added (resulting in pH <2). The samples were incubated 45 min at +37°C and then centrifuged (16,900 g, 10 min, +4°C). Twenty-five microliters of the resulting supernatant of each sample was carefully transferred to 0.5 mL Protein LoBind Tubes (Eppendorf AG). A C-terminally isotopically labeled peptide, containing U-13C6, U-15 N4-arginine [R] (aa 18-30, ADQLADESLEST[R]) was supplied by Sigma-Aldrich at over 95% peptide purity as determined by reversed phase HPLC. The peptide was dissolved and diluted in 0.1% aqueous FA to a final concentration of 5 fmol/μL. A 25 μL aliquot of the reference peptide was added to each immunoprecipitated brain homogenate sample after digestion and centrifugation.

#### CSF

The dried immunoprecipitated CSF samples were dissolved in 5 μL of a mixture of five isotopically labeled peptides, containing U-13C6, U-15 N4-arginine [R], U-13C6, U-15 N1-leucine [L] or U-13C6, U-15 N2-lysine [K] and common for the N-terminal part of both SNAP-25A and SNAP-25B. The peptide standard supplied by Sigma Aldrich (aa 18-30, see above) was mixed with four HeavyPeptide FasTrack 1 standards (Thermo Fisher Scientific) (~0.5 mg dissolved in 1 mL MilliQ water) (aa Ac2-16, AEDADMRNE[L]EEMQR; aa 17-31, RADQ[L]ADESLESTRR; aa 18-31 ADQLADESLEST[R][R]; and aa 32-40 MLQLVEES[K]) and diluted in 50 mM NH_4_HCO_3_ to a final concentration of ~100 fmol/μL (aa 18-30) and ~3 ng/μL (FasTrack 1 standards). Reduction of disulfide bonds (5 μL 10 mM dithiothreitol), alkylation (5 μL 10 mM iodoacetamide), and digestion (5 μL 5 mg/L trypsin) were performed as described above. To stop the enzymatic activity 4 μL 10% aqueous FA was added. The samples were centrifuged (16,900 g, 10 min, +4°C) and 20 μL of each sample was transferred to LC-vials (SUN-SRi).

### SRM-MS analysis of immunoprecipitated SNAP-25 from brain tissue

Aliquots (25 μL) of the 1:1 mixtures of stable isotope-labeled standard peptide and immunoprecipitated SNAP-25 were transferred to LC-vials (SUN-SRi) and analyzed by SRM-MS using an Accela 1250 pump (Thermo Fischer Scientific) coupled to a triple quadrupole mass spectrometer (TSQ Vantage, Thermo Fischer Scientific) with an IonMax source and HESI-II electrospray probe (Thermo Fischer Scientific). Mobile phases were 0.1% aqueous FA (v/v) (A) and 0.1% FA in 84% ACN in water (v/v) (B). Samples (20 μL) were loaded directly onto a Hypersil Gold-C18 column, (length 50 mm, inner diameter 2.1 mm, particle size 5 μm [Thermo Fischer Scientific]) with 0.1% aqueous FA at 100 μL/min. After 2 min of loading, the peptides were eluted off the column using the following linear gradient steps: 0 min 0%B; 4 min 17%B; 12 min 23%B; 15 min 100%B. The global MS parameters were: positive ion mode; spray voltage 3.5 kV; vaporizer temperature +350°C; sheath gas pressure 40 psi; auxiliary gas pressure 25 arbitrary units; capillary temperature +350°C; collision gas pressure 1.9 mTorr. Pinpoint software version 1.3.0 (Thermo Fischer Scientific) was used for method optimization and data processing.

### LC–MS/MS analysis of immunoprecipitated SNAP-25 from brain tissue

Aliquots (25 μL) of the 1:1 mixtures of stable isotope labeled standard peptide and immunoprecipitated SNAP-25 protein were transferred to LC-vials (Waters) and analyzed by LC-MS/MS. The LC-MS/MS spectra were acquired with a electrospray–linear quadrupole ion trap–Fourier transform ion cyclotron resonance (ESI-LQIT–FTICR) mass spectrometer equipped with a 7 T magnet (LTQ FT Ultra, Thermo Fischer Scientific) coupled to a multi-dimensional nanoflow chromatography system (Ettan MDLC, GE Healthcare). A Zorbax 300 SB-C18 trap column (length 5 mm, inner diameter 0.3 mm, particle size 5 μm [Agilent Technologies]) was used for on-line desalting and a reversed phase Zorbax 300 SBC18 column (length 150 mm, inner diameter 0.075 mm, particle size 3.5 μm [Agilent Technologies]) was used for high-resolution separation. Mobile phases were 0.1% aqueous FA (v/v) (A) and 0.1% FA in 84% ACN in water (v/v) (B). The separation was performed at a flow rate of approximately 250 nL/min by applying a linear gradient of 0-60% B for 50 min. The LTQ FT Ultra was set to acquire positive ions and operated in the data-dependent mode, where a scan cycle consisted of one full scan mass spectrum (m/z 350-1500) acquired in the FTICR mode (resolution 25,000), followed by tandem mass spectrometry (MS/MS) scans acquired in LQIT mode using collision-induced dissociation (CID) and wideband activation. Dynamic exclusion was enabled with repeat count 2 and exclusion duration 120 s. Isolation width was 3 m/z units, and the normalized collision energy to 35. Each scan consisted of three microscans.

### Database search and bioinformatic analysis

Database searches were submitted to the in-house database server by using Mascot Deamon 2.3.0 (Matrix Science). Database search parameters were; database (UniProtKB_Human 131030), taxonomy (Homo sapiens), enzyme (trypsin), variable modifications (acetyl [N-term], oxidation [M], Label:13C(6)15 N(2) [K], and Label:13C(6)15 N(4) [R]), fixed modifications (Carbamidomethyl [C]), mass values (monoisotopic), peptide mass tolerance (5 ppm), fragment mass tolerance (0.5 Da), and max missed cleavages (2).

### Quantification of high mass accuracy precursor ions

Peak detection and integration were performed using DeCyder 2.0 (GE Healthcare) using processing parameters as described in [42]. In-house developed software (Sequence and PeakExtractor) was used for *in silico* digestion and automatic peak mass matching.

### Top-down-LC-MS/MS analysis of intact SNAP-25 fragments from brain tissue

Soluble SNAP-25 forms immunoprecipitated with SMI81 from three different brain homogenate fractions were pooled in a 0.5 mL Protein LoBind Tube (Eppendorf AG) and dried in a vacuum centrifuge. The sample was dissolved in 25 μL of 0.1% aqueous FA for 1 h and then centrifuged (16,900 g, 10 min, +4°C). The LC-MS/MS spectra were acquired with the Ettan MDLC/LTQ FT Ultra system using the same LC settings and columns as for the digested samples (see above). The LTQ FT Ultra was operated in data-dependent mode with a scan cycle consisting of one full scan mass spectrum acquired in FTICR mode (m/z 500-1,200, resolution 50,000), and one MS/MS scan in FTICR mode (resolution 50,000) using CID. Each scan consisted of three microscans. Isolation width was 7 m/z units, and the normalized collision energy 35. To ensure MS/MS acquisition for the peptides of interest, an inclusion mass list was utilized. Peak picking and charge deconvolution of acquired spectra were performed using Mascot Distiller (Matrix Science) with processing parameters as described in [42]. Database search parameters were; database (UniProtKB_Human 120809), taxonomy (Homo sapiens), enzyme (none), variable modifications (oxidation [M]), fixed modifications (acetyl [N-term], carbamidomethyl [C]), mass values (monoisotopic), peptide mass tolerance (10 ppm), fragment mass tolerance (50 mmu), and instrument type (FTICR CID Distiller, meaning only singly charged fragment ions were considered in the database search).

### HR-SIM-MS analysis of immunoprecipitated SNAP-25 from CSF

High-resolution selected ion monitoring (HR-SIM) analyses were performed on a quadrupole–orbitrap mass spectrometer Q Exactive (Thermo Fisher Scientific) coupled to an Ultimate 3000 chromatography system (Thermo Fisher Scientific). The samples (15 μL) were loaded directly onto a Hypersil Gold-C18 column, (see SRM-MS analysis) with 0.1% aqueous FA at 100 μL/min. Mobile phases were the same as in the SRM-MS analysis. After 2 min of loading, the peptides were eluted off the column using the following linear gradient steps: 0 min 0%B; 4 min 13%B; 30 min 17%B; 50 min 26%B; 52 min 90%B. The IonMax ion source settings were: spray voltage, +4100 V; capillary temperature, +320°C; sheath gas pressure, 25 arbitrary units; auxiliary gas pressure, 10 arbitrary units; and heater temperature, +300°C. The instrument was set to acquire scheduled pairs of SIM scans and subsequent all ion fragmentation scans in profile mode allowing simultaneous detection of both the SNAP-25 peptide and the corresponding isotopically labeled peptide standard. The settings were common for both scans types and were as follows: resolution, 70,000; AGC target, 3e6; maximum injection time, 300 ms. Data acquisition and analysis were performed with Xcalibar software version 2.2 SP1.48 (Thermo Fisher Scientific) and Pinpoint 1.3.0. SNAP-25 levels for the different tryptic peptides were compensated for the different CSF volumes and reported as the ratio between the peak areas of the endogenous peptide and the labeled peptide standard multiplied by 10,000.

### Investigation of reproducibility

Approximately 5 pmol each of two isotopically labeled N-terminal SNAP-25 peptides (aa Ac2-16, AEDADM[R]NELEEMQ[R] and aa Ac2-20 AEDADMRNE[L]EEMQRRADQ, FasTrack 1 [Thermo Fisher Scientific]) were added to 15 mL CSF. The spiked CSF pool was divided into 890 μL aliquots, immunoprecipitated with SMI81 and digested as described above. HR-SIM-MS analysis of eight of the samples was performed on 8 μL injections. The CV of the measured levels was less than 10% (Additional file 1: Table S3).

### Statistical analysis

Because the distributions of most analytes were not normal (Shapiro-Wilk test, P <0.05), non-parametric statistics were used for analysis. Data are given as median (inter-quartile range). Differences between more than two groups were assessed with Kruskal-Wallis test. Statistically significant results (P < 0.05) were followed by Mann-Whitney U-tests to investigate group differences. Since there were no significant alterations of the levels of novel SNAP-25 biomarkers, Aβ1-42, and P-tau_181_ between the different cohorts, it was possible to perform the receiver operating characteristic (ROC) curve analysis and assess correlations in all patients with Alzheimer’s disease and controls. ROC curves were performed on each subject group on the tryptic peptides of SNAP-25 in order to assess their diagnostic value. For each tryptic peptide of SNAP-25 the area under the curve and a 95% confidence interval was calculated using GraphPad Prism 5. The correlation coefficients (rho) were calculated using the Spearman two-tailed correlation test. SPSS 20.0 was employed for most of the statistical analyses.

### Ethics

The present study was approved by the Regional Ethics Committee at the medical faculty Mannheim, University of Heidelberg, Germany and Lund University, Gothenburg University, Sweden. All patients gave their informed consent for research, which was conducted in accordance with the Helsinki Declaration The ethical principles abided by Netherlands Brain Bank are found at the website: (http://www.hersenbank.nl).

## Electronic supplementary material

Additional file 1: Table S1: Clinical and demographic characteristics of the brain tissue material. **Table S2.** Soluble SNAP-25 forms identified by LC-MS/MS. **Table S3.** HR-SIM-MS peak area of the human CSF tryptic SNAP-25 peptide Ac-2-16, and the spiked in isotopically labeled peptides Ac-2-16[R] and Ac-2-16[L]. **Figure S1.** Heat map for the relative signal intensities of individual tryptic peptides from SNAP-25B. **Figure S2.** Tandem mass spectra of three different soluble N-terminal fragments of SNAP-25 acquired in the FTICR mode. (PDF 658 KB)

## Abbreviations

Aβ1-42: Amyloidβ1-42
CSF: Cerebrospinal fluid
CID: Collision-induced dissociation
FA: Formic acid
FTICR: Fourier transform ion cyclotron resonance
HR-SIM-MS: High resolution–selected ion monitoring–mass spectrometry
LQIT: Linear quadrupole ion trap
LC-MS/MS: Liquid chromatography–tandem mass spectrometry
MCI: Mild cognitive impairment
MMSE: Mini-mental state examination
ROC: Receiver operating characteristic
SRM-MS: Selected reaction monitoring–mass spectrometry
SNARE: Soluble N-ethylmaleimide sensitive fusion attachment protein receptor
SNAP-25: Synaptosomal-associated protein 25
P-tau_181_: Tau phosphorylated at threonine 181
T-tau: Total tau.
