# Supplementary material for: SNAP-25 is a promising novel cerebrospinal fluid biomarker for synapse degeneration in Alzheimer’s disease
Source: Mol Neurodegener. 2014 Nov 23;9:53. doi: 10.1186/1750-1326-9-53 (PMC4253625; doi:10.1186/1750-1326-9-53)
Supplement: Supplementary file 1 — Additional file 1: Table S1: Clinical and demographic characteristics of the brain tissue material. Table S2. Soluble SNAP-25 forms identified by LC-MS/MS. Table S3. HR-SIM-MS peak area of the human CSF tryptic SNAP-25 peptide Ac-2-16, and the spiked in isotopically labeled peptides Ac-2-16[R] and Ac-2-16[L]. Figure S1. Heat map for the relative signal intensities of individual tryptic peptides from SNAP-25B. Figure S2. Tandem mass spectra of three different soluble N-terminal fragments of SNAP-25 acquired in the FTICR mode. (PDF 658 KB) [file 13024_2014_561_MOESM1_ESM.pdf]

SUPPLEMENTARY INFORMATION

Supplementary Tables 1-3 and Figures 1-2

SNAP-25 is a promising novel cerebrospinal fluid biomarker for synapse degeneration in Alzheimer's disease.

Ann Brinkmalm[1], Gunnar Brinkmalm[1], William G. Honer[2], Lutz Frölich[3], Lucrezia Hausner[3], Lennart Minthon[4,5], Oskar Hansson[4,5], Anders Wallin[1], Henrik Zetterberg[1,6], Kaj Blennow[1*], and Annika Öhrfelt[1]

[1]*Institute of Neuroscience and Physiology, Department of Psychiatry and Neurochemistry, Sahlgrenska Academy at the University of Gothenburg, S-431 80 Mölndal, Sweden*

[2]*Department of Psychiatry, University of British Columbia, Vancouver, Canada*

[3]*Department of Geriatric Psychiatry, Central Institute for Mental Health Mannheim, University of Heidelberg, Mannheim, Germany*

[4]*Clinical Memory Research Unit, Department of Clinical Sciences, Lund University, Sweden*

[5]*Memory Clinic, Skåne University Hospital, Sweden*

[6]*UCL Institute of Neurology, Queen Square, London WC1N 3BG, United Kingdom*

Supplementary Table 1 Clinical and demographic characteristics of the brain tissue material.

Diagnosis	Numbers (Female/Male)	Age at death [years][a]	Post-mortem delay [min][a]
Controls	15 (7/8)	73 (70-77)	340 (330-383)<br>$p<0.05$)
Alzheimer's disease	15 (6/9)	73 (71-76)	300 (268-330)

[a]Data are given as median (inter-quartile range) There is no correlation between post-mortem delay and levels of SNAP-25, 18-30.

Supplementary Table 2 Soluble SNAP-25 forms identified by LC-MS/MS.

SNAP-25 form	Theoretical mass [Da][a]	Mass deviation [ppm]	Fragments detected (b/y)
Ac-2-20	2348.0132	0	12/13
Ac-2-21	2461.0961	2	11/10
Ac-2-22	2532.1332	3	9/10
Ac-2-30	3449.5423	3	6/10
Ac-2-32	3736.6838	1	11/9
Ac-2-33	3849.7679	2	15/16
Ac-2-46	5276.5095	2	13/7
Ac-2-47	5389.5936	1	14/7

[a]monoisotopic mass values

Supplementary Table 3 HR-SIM-MS peak areas[a] of the human CSF tryptic SNAP-25 peptide Ac-2-16, and the spiked in isotopically labeled peptides Ac-2-16[R] and Ac-2-16[L].

Aliquot\peptide	Ac-2-16		Ac-2-16[L]		Ac-2-16[R]	
	3+	2+	3+	2+	3+	2+
#1	1.9	0.67	50	22	52	19
#2	1.1	0.42	36	17	42	15
#3	1.3	0.49	43	19	45	17
#4	1.6	0.55	44	20	49	18
#5	1.3	0.41	41	18	44	15
#6	1.4	0.46	43	18	48	17
#7	1.2	0.43	40	17	43	15
#8	1.5	0.51	43	19	44	16
Average	1.4	0.49	43	19	46	17
StDev	0.25	0.09	3.9	1.9	3.4	1.5
CV[%]	18	18	9.1	10.1	7.3	9.0

[a]presented value is the Pinpoint peak area divided by 10^5

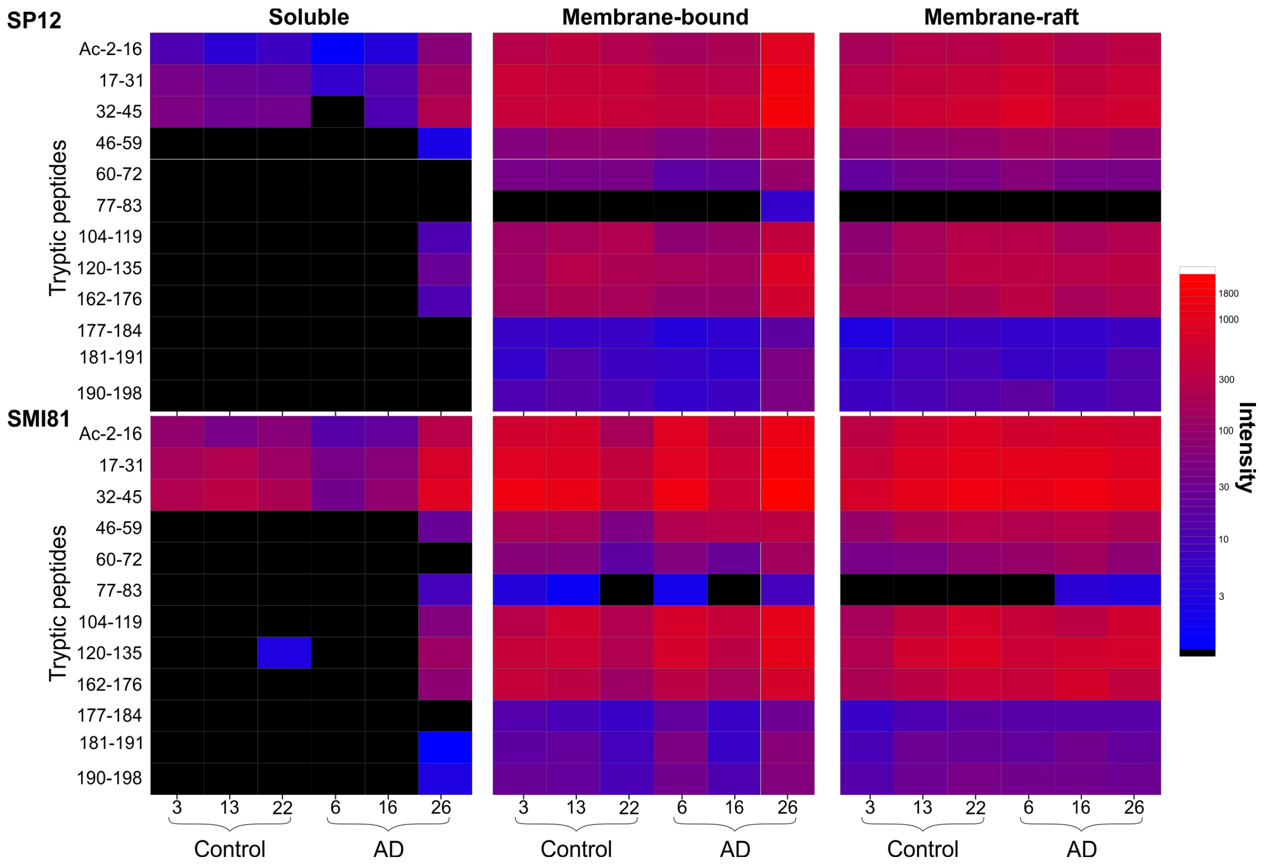

Supplementary Figure 1 Heat map for the relative signal intensities of individual tryptic peptides from SNAP-25B. Biochemically fractionated soluble (left panels), membrane-bound (middle panels) and membrane-raft associated (right panels) proteins of superior parietal gyrus from controls (N=3) and patients with AD (N=3) were immunoprecipitated with SP12 (upper panels) and SMI81 (lower panels) respectively. LC-MS/MS ion chromatograms of all peptides identified as belonging to SNAP-25B were extracted. An increase of signal intensity is seen as a white shift.

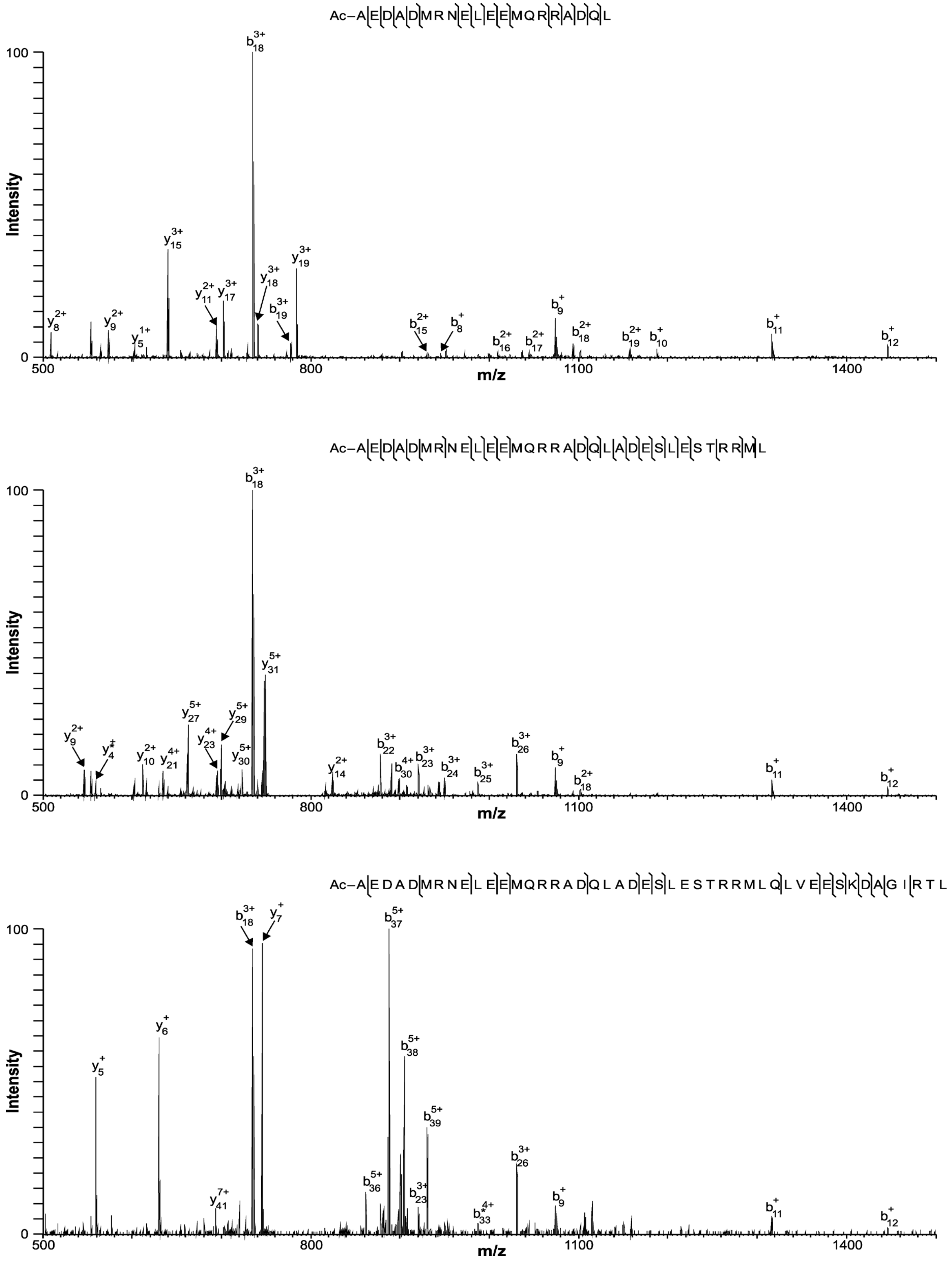

Supplementary Figure 2 Tandem mass spectra of three different soluble N-terminal fragments of SNAP-25 acquired in the FTICR mode. Fragments confirmed by database search are indicated in the respective sequences.
